# Biases arising from linked administrative data for epidemiological research: a conceptual framework from registration to analyses

**DOI:** 10.1007/s10654-022-00934-w

**Published:** 2022-11-05

**Authors:** Richard J. Shaw, Katie L. Harron, Julia M. Pescarini, Elzo Pereira Pinto Junior, Mirjam Allik, Andressa N. Siroky, Desmond Campbell, Ruth Dundas, Maria Yury Ichihara, Alastair H. Leyland, Mauricio L. Barreto, Srinivasa Vittal Katikireddi

**Affiliations:** 1 MRC/CSO Social and Public Health Sciences Unit, University of Glasgow, Berkeley Square, 99 Berkeley Street, Glasgow, G3 7HR UK; 2 UCL Great Ormond Street Institute of Child Health, UCL, London, UK; 3 Centro de Integração de Dados e Conhecimentos para Saúde (Cidacs), Fundação Oswaldo Cruz, Salvador, Brazil; 4 Faculty of Epidemiology and Population Health, London School of Hygiene and Tropical Medicine, London, UK; 5 Departamento de Estatística, Universidade Federal do Rio Grande do Norte, Natal, Brazil; 6 Instituto de Saúde Coletiva, Universidade Federal da Bahia, Salvador, Brazil

**Keywords:** Record linkage, Linkage error, Data linkage, Administrative data, Epidemiological biases

## Abstract

Linked administrative data offer a rich source of information that can be harnessed to describe patterns of disease, understand their causes and evaluate interventions. However, administrative data are primarily collected for operational reasons such as recording vital events for legal purposes, and planning, provision and monitoring of services. The processes involved in generating and linking administrative datasets may generate sources of bias that are often not adequately considered by researchers. We provide a framework describing these biases, drawing on our experiences of using the 100 Million Brazilian Cohort (100MCohort) which contains records of more than 131 million people whose families applied for social assistance between 2001 and 2018. Datasets for epidemiological research were derived by linking the 100MCohort to health-related databases such as the Mortality Information System and the Hospital Information System. Using the framework, we demonstrate how selection and misclassification biases may be introduced in three different stages: registering and recording of people’s life events and use of services, linkage across administrative databases, and cleaning and coding of variables from derived datasets. Finally, we suggest eight recommendations which may reduce biases when analysing data from administrative sources.

## Introduction

Analyses of linked administrative data, especially electronic health records, are transforming epidemiology. Useful data sources for epidemiological research include civil registration systems, which record vital events, such as births, deaths and marriages [1], and records generated during the provision of health and social care. Administrative datasets are often substantially larger than those collected for research purposes, such as surveys, trials, panel studies and longitudinal birth cohorts. Additionally, administrative datasets are more inclusive of people less likely to respond to surveys, facilitate follow-up with limited attrition and can provide directly relevant evidence for policy making [2]. These features make the use of administrative data for research attractive, but their use presents many challenges. Decisions around whose data are collected, what information is recorded and how it is coded will be motivated primarily by the operational purposes for data collection [3]. While analysts’ ability to influence these processes is limited, these and other data processing steps require consideration if biases are to be mitigated. Depending on how administrative processes define analytic samples, registration and recording processes may introduce selection, information or collider biases [4–6].

The quality of administrative data will be determined by practical constraints. Data quality will be affected by the purpose for which data are collected and the requirements and incentives placed upon those responsible for data entry. For example, death certificates may not be accurately completed by doctors in less well-resourced areas [7].

Administrative data sources are typically created by a single system covering a narrow domain of information [3]. For example, hospital care records will be available from healthcare providers, while educational qualifications will be available from education departments. However, health is the product of multiple determinants [8], therefore, linkage across datasets from different domains is often required. Nevertheless, amenability to linkage is often not considered by data producers, with a unique identifier not available across datasets in most countries. Consequently linking datasets may not be straightforward and often involves applying matching/linkage algorithms [2]. In many settings, straightforward deterministic linkage algorithms are applied to administrative data, which look for agreement on a set of partial identifiers such as name, date of birth, and postcode. More sophisticated probabilistic linkage methods may also be used, which involve generating match weights representing the likelihood that two records belong to the same individual or entity, based on agreement patterns across a set of partial identifiers [9]. In general, linkage is performed by a trusted organisation, meaning that researchers do not have access to identifiable data.

The quality of linkage algorithms may be quantified in terms of the proportions of true matches, true non-matches, missed links and false links [10]. Linkage accuracy may vary with the quality or completeness of data and this in turn may be related to variables of research interest. For example, there may be more misspellings for members of ethnic minority groups as registration clerks may be unfamiliar with their names [11, 12]. There are existing reporting guidelines including GUILD aimed at data providers [13] and RECORD aimed at researchers [14], and guides for estimating quality within pairwise [6, 10] and cluster linkages [15]. Increasing access to administrative data for researchers and related ethical issues have been prioritised in literature [16, 17]. We complement these studies by looking at the whole data ecosystem and investigating where biases could arise.

Since epidemiologists typically have limited scope to influence administrative data collection or linkage, the implications of potential biases arising from the use of linked data need to be appreciated [18]. We present a conceptual framework to illustrate the processes through which biases might arise, illustrated by our experiences in analysing the 100 Million Brazilian Cohort (100MCohort—See Barreto et al. [19] and Box 1). We conclude with recommendations to help identify and reduce biases in future data linkage studies.

Box 1: The 100 million Brazilian cohort The 100MCohort was created by the Centre for Data and Knowledge Integration for Health (Centro de Integração de Dados e Conhecimentos para Saúde—CIDACS) at Oswaldo Cruz Foundation (Salvador, Brazil) to investigate the socio-determinants of health and evaluate the impact of social policies on health [19, 20]. The baseline of the cohort consists of 131 Million people whose families have applied for social benefits from the federal government between 1st January 2001 and 31st December 2018 and have therefore been registered in the Cadastro Único para Programas Sociais (CadÚnico) database. The cohort includes 62% of the population [21], with over-representation of the more socially disadvantaged people, for example only 52% of mothers giving birth in the cohort have more than 7 years of education compared to 69% of all mothers giving in birth in Brazil registered in the Live Birth Information System (SINASC) [22].

The CadÚnico contains a wide variety of sociodemographic information such as age, sex, family composition, income, education, housing type, and utility supplies [23]. A unique identifier, the Social Identification Number (NIS) exists in CadÚnico and databases for various administered social programmes, including Bolsa Família (PBF—a conditional cash transfer programme) and Minha Casa, Minha Vida (a housing programme) [23] which therefore allows exact deterministic linkage. However, linkage to health datasets is more challenging since no common unique identifier keys are available across health and social programme databases [23]. Linkage has therefore been carried out using CIDACS-RL (Centre for Data and Knowledge Integration for Health—Record Linkage), an iterative two stage linkage algorithm that first performs exact matching usually based on five attributes (name, mother’s name, date of birth, gender, and municipality of residence), followed by a second stage where matching is based on a score (0 to 1) built from the same variables [24]. Health databases that are linked include the Mortality Information System (SIM) (2000 onwards), the Hospital Information System (SIH) (2000–2017) and the disease notification information system (SINAN) (2001 onwards). More detail on the databases and linkage processes has been published [21, 23, 24].

For the purposes of illustration, we focus primarily on PBF which has been highly successful in alleviating poverty [25]. It provides a per capita monthly income for families living with an income below the poverty line, with a stipend being provided for each child (up to a total of ten) under the age of 16, and a slightly higher amount for 16-or 17-year-old children, provided the children attend school and use health services. PBF is conditional on pregnant women receiving care, and children receiving vaccinations and growth monitoring, and attending school. Conditions are monitored with the aim of helping participants rather than punishing them, with the programme generally being viewed positively by participants [26]. As receipt of PBF is one of the major incentives for registering with CadÚnico (approximately 80% of those who register eventually receive it), it is important to understand the principles underpinning Bolsa Família and its administration.

In addition to alleviating poverty, PBF aims to increase women’s control of household resources by preferentially paying money to women [25]. It is administered as a partnership between federal and local government, with the federal government financing the policy but administration conducted by municipalities (5,565 areas across Brazil that range in population size from 776 to more than 11 million). Each municipality has a quota of households roughly determined by poverty levels. If the number of households applying for PBF is below the quota then all eligible applicants will receive the benefit [25]. The result is that not only are there likely to be systematic differences between those who receive PBF, and who does not, there are also geographic differences in receipt [25]. Using the 100MCohort the impact of PBF has been estimated on different health outcomes [27–30] among other applications [10].

## A conceptual framework for biases arising through data linkage processes

In this section, we present a framework to describe sources of bias arising from linked administrative analyses and demonstrate how this framework can be harnessed to explore biases.

**Fig. 1:**
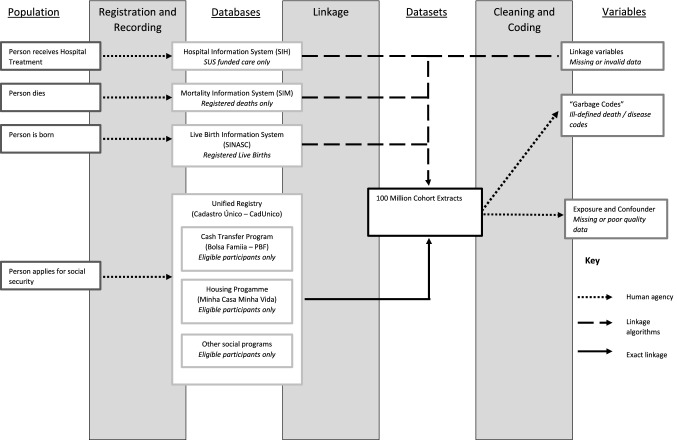
A framework describing the data generation processes for linking administrative data, illustrated by the 100 Million Brazilians cohort

Figure 1 describes the processes used to generate datasets based on the 100MCohort. Events and people (far left column) are recorded in administrative databases (centre left) from which analytical datasets are derived. These contain variables for analysis (far right column) which can be used to describe population characteristics and assess exposure-outcome relationships. In practice, registration and recording of people and events may not happen, errors in the linkage process may generate missed and false links, and there may be systematic differences in data quality that cannot be addressed by cleaning and coding of available data. All these processes can introduce biases.

Different parts of the data flow can introduce biases (indicated by different types of arrows in Fig. 1): first, data recording is dependent on decisions made by policy makers, administrators and people using services; second, linkages are carried out using linkage algorithms; and third, cleaning and coding of variables is carried out by data scientists responsible for linkage, as well as the analysts themselves.

## Recording and registration

People and events relevant to answering research questions may not always be recorded resulting in “missing records”. Reasons for missing records include people not engaging with services or registration processes because they face barriers such as racism, homelessness or living in remote areas, or data processing errors. We will illustrate this with three examples: the recording of hospital treatments in the Hospital Information System (SIH), ascertainment of mortality in the Mortality Information System (SIM) and the registration and updating of records in the Unified Register for Social programs (CadÚnico).

The SIH in Brazil only includes publicly funded healthcare (provided by the Sistema Unico de Saude—SUS) and some Brazilians fund their care through other means, with 27.9% of the Brazilian population having some form of private health insurance in 2013. While most Brazilians rely on SUS [31] it accounts for less than half of all healthcare spending [32]. Many high-income patients commonly use private sector services but switch to SUS for more complex and costly operations, while low-income patients are much more likely to use SUS in general. Consequently, there is a relationship between socioeconomic position and the type of healthcare received. Relying on SIH for health care data may under-report health conditions and receipt of medical treatment of people in the general population, particularly for elective healthcare and in socioeconomically privileged groups. In addition, we expect higher coverage for more complex procedures.

Brazil aims to ensure all deaths are registered in SIM and recent studies show that registration is generally high but levels vary substantially; for example, around 98% of deaths were estimated to have been registered overall in 2016, although this was as low as 93% in some states [33]. There are geographic and social variations in the completeness of death registrations, with higher under-registration in the most socio-economically disadvantaged areas [33]. Consequently, death rates for people living in disadvantaged areas are likely to be underestimated.

The 100MCohort dataset is based on the CadÚnico (Box 1) which only includes people, and their family members, who have applied for, but not necessarily received, social assistance. This defines the denominator for any analysis, covering approximately 62% of the Brazilian population [19]. This would introduce selection biases if the population required to address a research question includes people who have not applied for benefits and so are not in the dataset. Active registration processes help mitigate biases that might occur because they actively recruit members of vulnerable groups who might otherwise not engage with services. Refining research questions so that estimated parameters relate to the population for which data are available and estimating parameters, such as the average treatment effects among the treated, which require weaker assumptions [34], are other ways in which biases can be mitigated.

**Fig. 2:**
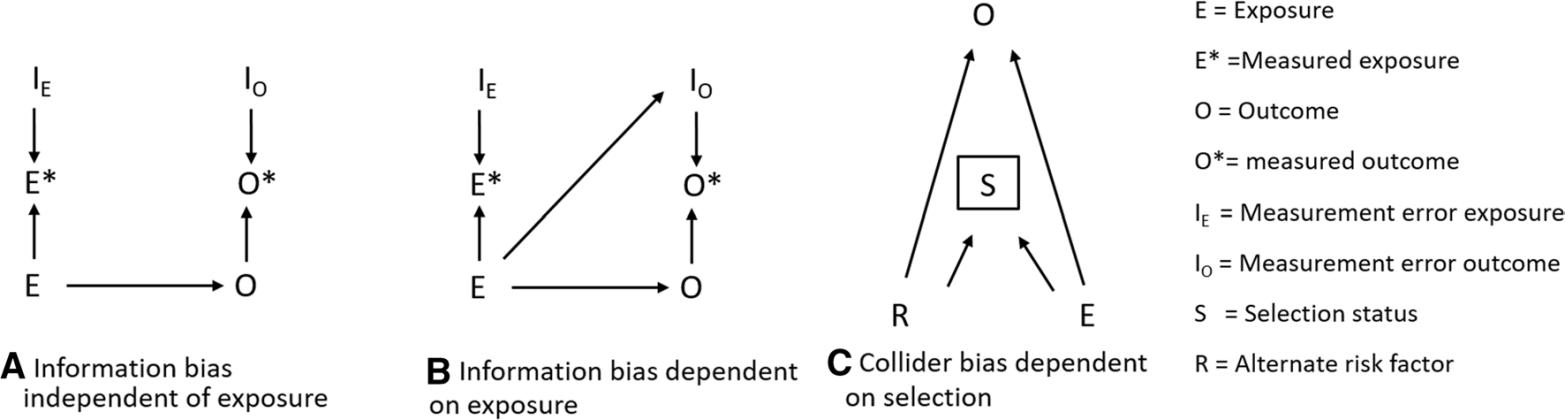
DAGs for information bias both independent and dependent on exposure and a DAG for collider bias

Factors influencing eligibility and motivation to apply for services covered by administrative databases are potential sources of bias. For example, people receiving Programa Bolsa Família (PBF—a conditional cash transfer programme—see Box 1) will tend to have larger families and lower incomes than the general population, both of which are generally associated with poorer health. In addition, any analyses using the 100MCohort are effectively conditioning on membership of CadÚnico, thus cohort membership acts as a collider and can generate collider biases [35]. In Fig. 2c exposure E (e.g. application for PBF) is a determinant of CadÚnico membership (S in box) which is a conditioning variable. Thus CadÚnico membership acts as a collider opening pathways between the outcome (O) and any risk factors (R—such as application for Minha Casa, Minha Vida, a housing programme) which are related to CadÚnico membership, and thus potentially introducing confounding.

Systematic differences in updating of records may also introduce biases. For example, active recipients of benefits will have their records updated in CadÚnico every two years, while this will not necessarily occur for those no longer receiving benefits. Using updated records may lead to differential rates in linkage errors such as false and missed links (see below) dependent on receipt of benefits, which for many research questions will be the exposure of interest (see Fig. 2b). In contrast, the assumption of non-differential linkage errors between the two groups may be more plausible for the initial records (see Fig. 2a and Hernán and Cole [36] for a fuller discussion). Thus, in some circumstances it may be better to use initial records that are not updated to reduce systematic biases, even if this results in poorer linkage accuracy. There may also be systematic differences in whether or not initial records (prior to updating) are accurate. Municipality of residence is among the linkage variables used for the 100MCohort. If people move and their municipality is not updated there is a risk of poorer linkage. This is pertinent given some policies of interest, such as PBF, are associated with reduced migration [37] which is in turn associated with poorer linkage accuracy and hence might lead to information bias (Fig. 2b).

## Linkage

The second source of bias relates to linkage errors, which can lead to individual records either not being linked (missed links) or being falsely linked. Missing links can compound the problems caused by missing records, and are more likely to arise when there are quality issues or missing data for variables that are used in the matching process. A key factor that helps to estimate linkage accuracy is whether or not all records are expected to be linked. We will illustrate this with two different linkages carried out on the 100MCohort in relation to birth and deaths.

The Live Birth Information System (SINASC) was linked to the baseline records for the 100MCohort for the years 2001 to 2015 [38]. Identification of missed links was possible because while at that time only 57% of population is covered by the 100MCohort, it can reasonably be assumed that all infants born in Brazil should have records in SINASC. Thus, any unmatched 100MCohort records for infants can be assumed to be either missed links or failures in the birth registration system. Between 2001 and 2015 the percentage of birth records successfully linked with the 100MCohort increased from 39.4% to 80.2%. People for whom records were linked to the cohort tended to be better educated, living in urban areas, had better living conditions, and were more likely to be White. Mortality records in SIM were also linked to 100MCohort for the period between 2001 and 2015, during which time the number of people registered in 100MCohort increased from 325,633 to 114,007,317. The average linkage rate for this period is 17.3%. However, because neither SIM nor 100MCohort fully enumerate the population, this figure is not informative about the extent of missed links.

A second form of linkage errors are false links. False links may leave no direct traces in matched records and identifying them is very challenging. One option is to identify logically implausible values e.g., hospital readmission after death [39]. However, false links for logically implausible values will only represent a subset of possible false links. Other options require researchers to have access to identifiable data or greater engagement with those carrying out the linkage than is typical. For a limited number of records, it may be possible to go through individual linkages and check for possible inconsistencies. The CIDACS-RL linkage algorithm was assessed by carrying out a linkage between CadÚnico (114,008,179 individual records) and a tuberculosis notification dataset (1,182,777 case records) [24]. The optimal linkage cut point (the linkage score based on similarity that had to be equalled or exceeded for records to be considered linked) was assessed by taking a 30,000 subset of potentially linked pairs and manually verifying the sample. Using the optimal cut point with the 30,000 sample of potentially linked pairs, 42.5% were considered matches, and of these, 8.9% were found to be false matches. However, the optimal cut point appeared to miss 7.5% of true matches [24].

## Cleaning and coding

A crucial stage in addressing potential biases with administrative data is cleaning and coding, not only of analytical variables but also variables used for linkage. An example of data quality issues for analytical variables is ill-defined causes of death (IDCD) [7], i.e., codings that are not informative as to the cause of death. For research questions addressing all-cause mortality, IDCDs are not directly problematic, although they could indicate problems relating to data collection. IDCDs pose more of a problem for research questions related to cause-specific deaths, as there can be systematic differences in the proportions of IDCDs. For example, in the period 1998 to 2012, the proportion of IDCDs in the north of Brazil was 14%, while the proportion of IDCDs was only 5% in the South [40]. Overall, the proportion of ICDS has been decreasing over time.

Linkage variables are an important subset of variables affected by poor data quality (e.g., due to miscoding or missingness) as data quality may indicate groups of people who are at greater risk of missed links. In the 100MCohort the linkage variables include name, sex, mother’s name, municipality and date of birth. Linking records in the first year of life is particularly challenging as many births are not recorded with valid names; for example, of the 3,013,228 records in SINASC for 2015, the majority (2,609,537) had invalid baby names [24].

Geocoding individual level records (by linking zip codes or census tracts, for example) is widely conducted in epidemiologic research to provide additional information, particularly when interested in environmental exposures (such as air pollution) or proxies for socioeconomic position (e.g., area-based deprivation). Ensuring accurate addresses when cleaning administrative data is crucial, as it has the potential to lead to mismatches, the consequences of which may be reduced sample sizes and increased selection and information biases [41].

## Conceptualising biases in linked data: an example from the 100 Million Brazilian cohort

Understanding biases that arise from using linked administrative data requires interrogating the data themselves, and drawing on knowledge about the administrative and linkage processes used to generate the data. Table 1 is an adaptation of a Johari window [42] and divides potential biases according to these two dimensions. However, information required to assess these biases may not always be available. For simplicity we present each dimension as dichotomous but in practice there may be gradations.

**Table 1:** Classification of potential biases arising from linkage of administrative data based on knowledge about data generating processes and data inspection. Examples from the 100MCohort dataset

Knowledge about processes	Knowledge about data	
	Known	Unknown
Known	*Missing values e.g. education data* The datasets provide information on who has missing values. Knowledge about the processes generating the data inform when and where in the system the failure to record appropriate values occurred	*Eligibility criteria for social security benefits* Knowledge about eligibility criteria is a useful source of information about who is not registered in the CadUnico, despite there being no data for non-registered people. This provides information about the population for which inferences can be made
Unknown	*Missed links between 100MCohort and SIM* Unmatched records provide data indicating that missed links may have occurred. However, as neither data sources fully covers a respective population, it is very difficult to quantify the extent of missed links	*Under ascertainment of mortality* It is a legal requirement that all deaths be registered. However, for various reasons this does not always occur. Estimating the extent of unregistered deaths requires using data and methods that are independent of the administrative systems

Consideration of the data availability and our knowledge about the generating process can help guide study design and interpretation of results. Knowledge about how the data has been processed combined with available data (top left) may allow statistical models to address sources of bias. For example, methods to reduce biases arising from missing values may be helpful, if observed covariates can be used to predict missed linkages [43, 44]. Knowledge about the processes generating the data can inform study design and how generalizable the results’ interpretations are, even if data allowing statistical solutions are unavailable (top right). Available data may indicate a problem exists, but lack of knowledge about the data-generating processes limits its usefulness (bottom left). Some solutions to bias may still be possible, such as restricting analyses to geographies with fewer missed links. However, depending on circumstances this may create different analytical problems such as reduced statistical power and generalizability. Finally, biases may exist that cannot be estimated or addressed based on the observed data or knowledge of the data-generating processes (bottom right). These biases might only be identified by comparison to other literature or external data sources.

## Discussion

The use of large administrative datasets for research has several advantages and limitations, which applies to the 100MCohort [45]. Such datasets may allow novel and powerful insights to be obtained but, as the data was designed for other than research purposes, correct inference and analyses utilising such data is difficult. Here we have presented a framework for conceptualising the sources of bias that may arise when using linked administrative data. Recording and registration processes, that define populations and outcomes, are potential sources of selection, collider and numerator biases. Missed and false links during the linkage process may lead to misclassification biases, with this risk increased when the quality of variables used for linkage is sub-optimal. We suggest that identification, mitigation and appropriate consideration of these biases can be achieved by both careful analysis of available linked data and detailed knowledge of the data-generating processes (including the linkage process). If parts of a well-defined sample can be assumed to have reasonably accurate linkage, an option is to restrict the analytic sample to this sample. Then using fairly strong assumptions, methods can be applied such as re-weighting the analytic sample so that it matches the target population [46], or multiple imputation can be used to create synthetic observations that replace those that have been excluded by restriction [47].

The legal and ethical implications of the use of large-scale administrative data sources for research are still under development. Debates are commonly focused on issues relating to confidentiality and consent. However, it is also in the public interest that research arising from administrative data can be validated. More discussion is required on balancing the availability of data to assess biases, against issues of confidentiality and for consent of personal data use. Deriving the necessary information to quantify biases may require retaining information from many different stages of the linkage process. In addition, extracting, processing and linking large datasets can be incredibly computationally intensive and time consuming, and so it may not be practical to repeat data processing for large and complex linkages. Thus, it is important that everybody who has some responsibility for data processing has sufficient awareness about potential biases to ensure that the correct balance is achieved between protecting privacy and mitigating the risks posed by unknown biases. This will require appropriate training for everybody involved in data processing, from data controllers through to the analysts interpreting the results. The information available to investigate possible biases may be improved if there is greater collaboration between the researchers carrying out the analyses and those conducting linkage. Historically, this has been difficult as data protection laws may be interpreted in ways that create barriers to information being shared. However, there are ways to share non-disclosive information that can help assess the provenance and quality of linked data [10, 45]. Guidelines are also available to direct what information should be shared and reported for linked administrative data analyses [13, 14].

The 100MCohort is an example of a highly developed linkage, and represents the potential of what can be achieved using existing resources in a middle-income country. Moreover, many of the challenges and biases presented in the paper in an abstracted form are similar to those occurring in more developed settings [36]. Sample selection is potentially an issue in the USA where even publicly funded care through Medicaid and the Children’s Health Insurance Program covers less than 30% of the population under the age of 65 [48]. While in countries such as Scotland where publicly funded healthcare data in theory enumerates the whole population, in practice coverage is only 98% [49] and among this population some additional care will be provided privately. Additionally, the 100MCohort is a considerable investment, and is built on decades of work to improve statistics in Brazil [50]. Thus, in some countries much work may be required to create the necessary data infrastructure for similar cohorts to be created.

This paper represents the critical reflections of a multidisciplinary team that has been developing analyses using the 100MCohort and other administrative data sources. We believe that our emphasis on considering the whole process when deriving datasets from linked administrative sources complements the existing literature. Previous studies have tended to focus on specific aspects such as statistical challenges [3], linkage errors [10] or assessing linkage quality [6]. Over the course of developing the 100MCohort, linkage rates have improved considerably, and the linkage rates between 100MCohort and health databases are similar to linkage rates achieved in comparable cohorts from high-income countries which varied from 89 to 95% [51, 52]. However, even small error rates can introduce biases, leading to systematic differences in successful linkage rates between groups as demonstrated by the 100MCohort and other studies [52, 53]. To minimise situations where these potential disturbances could exist, administrative data should be considered part of a triangulation process [54], or to help make decisions about whether use of more expensive methods and time consuming methods are suitable [55].

We hope that this paper encourages others to draw on their experiences with using linked administrative data and generate a debate aimed at improving best practices. We make eight recommendations which we believe will reduce biases that may arise when using linked administrative data:

1. Researchers should consider the process of generating linked administrative data for research in its entirety, when investigating possible sources of bias. This will require greater collaboration between analysts and those conducting the linkage.
2. In advance of carrying out any linkage, potential sources of selection and information biases should be identified. Tools that could help do this include creating an overall flow chart for the potential linkage (similar to Fig. 1), and project specific DAGs for information bias (see Fig. 2b and Hernán and Cole [36] and selection bias (see Fig. 2c and Hernán, Hernández-Díaz et al. [56]). Potential sources of bias for which data can be collected should be identified and indicators of linkage quality should be recorded as part of the linkage process. Sources of bias that exist in theory, but which have not been measured should be noted as a limitation. Biases may arise while extracting data from databases, and preparing individual datasets for linkage, and computational costs may mean that it is only practical to carry out the linkage process once. Thus, it may only be possible to collect the information necessary for describing biases, if this is planned for in advance. Organisations responsible for conducting data linkage should consider integrating the provision of information about potential linkage biases into their processes.
3. Data linkers, where possible, should provide information on the characteristics of unlinked records, the characteristics of unlinkable records (i.e., those with poor identifier data quality), and on the level of certainty with which each link has been made (e.g. match rank or probabilistic match weight) [13, 14, 45]. This information should help researchers to understand where potential biases might arise, and allow them to perform sensitivity analyses to assess the extent to which different matching rules might influence results.
4. A clear distinction is needed between internal validity, i.e., is the study well designed and the results essentially true, and external validity, i.e., will these results generalise to other studies and settings. If the aim of the analysis is to estimate causal effects, it may be better to restrict the analytical sample to areas or groups where the risk of biases is smaller, and there may be consequences for generalisability if statistical solutions are not available.
5. Parameters estimated using administrative data should be based on assumptions [5, 34, 57] that are informed by evidence, theory and available data. This may be more achievable for estimates that require weaker assumptions. For example, estimating the average treatment effect for the treated requires a weaker positivity assumption (i.e. that all patients have a non-zero chance of being exposed), than the average treatment effect for the whole population, which requires the whole population to have a non-zero chance of being exposed [58].
6. It will not be practical to investigate all possible sources of bias. Priority should be given to biases likely to have the biggest impact on the research question. In addition, some solutions may not have the same consequences for all population subgroups. For example, limiting generalisability by restricting analyses to urban, as opposed to rural, areas with better data quality, may or may not be appropriate depending on the research question.
7. Often information necessary to understand linkage biases is not available from organisations conducting linkage—barriers to accessing such information should be reduced. Training on the possible sources of bias arising from using linked administrative data needs to be extended to those responsible for ethical and legal oversight of administrative data and computer scientists carrying out the linkage.
8. To improve the quality of collected data, it is imperative that health professionals and administrators are aware of the importance of administrative data for research and informing policy [59]. Improved training in standardised data collection protocols and financial incentives have been shown to improve the quality of ethnicity records [60]. Consulting patient groups about the collection and governance of data may also improve the quality [60].

In conclusion, administrative data present many opportunities for epidemiological research, but maximising their potential requires possible sources of bias to be better understood and minimised.
